# Research Progress of Selenium-Enriched Foods

**DOI:** 10.3390/nu15194189

**Published:** 2023-09-28

**Authors:** Zhenna Chen, Yiqing Lu, Xiaoling Dun, Xinfa Wang, Hanzhong Wang

**Affiliations:** Key Laboratory of Biology and Genetic Improvement of Oil Crops, Oil Crops Research Institute of Chinese Academy of Agricultural Sciences, Ministry of Agriculture and Rural Affairs, Wuhan 430062, China

**Keywords:** selenium, selenium-enriched foods, physiological functions, supplementation, analysis of selenium and its species

## Abstract

Selenium is an essential micronutrient that plays a crucial role in maintaining human health. Selenium deficiency is seriously associated with various diseases such as Keshan disease, Kashin–Beck disease, cataracts, and others. Conversely, selenium supplementation has been found to have multiple effects, including antioxidant, anti-inflammatory, and anticancer functions. Compared with inorganic selenium, organic selenium exhibits higher bioactivities and a wider range of safe concentrations. Consequently, there has been a significant development of selenium-enriched foods which contain large amounts of organic selenium in order to improve human health. This review summarizes the physiological role and metabolism of selenium, the development of selenium-enriched foods, the physiological functions of selenium-enriched foods, and provides an analysis of total selenium and its species in selenium-enriched foods, with a view to laying the foundation for selenium-enriched food development.

## 1. Introduction

Selenium is an essential micronutrient and plays important roles in the normal physiological activities of a living organism [1]. In human organisms, selenium is metabolized as 25 identified selenoproteins, which have various biological functions such as antioxidant and anticancer effects, and improvement of fertility and reproduction [2]. Recent studies have shown that selenium deficiency can lead to various chronic diseases [3,4,5], and approximately one billion people are suffering from selenium deficiency [6]. Therefore, a daily intake of selenium is recommended to maintain human health [7]. It is worth noting that the bioactivity and bioavailability of selenium in humans depend on its chemical species, which include inorganic selenium (e.g., Se(VI) and Se(IV)) and organic selenium (e.g., methylselenocysteine (MeSeCys), selenocysteine (SeCys), selenocystine (SeCys_2_), and selenomethionine (SeMet)) [8]. Organic selenium is less toxic, and has greater bioactivity and higher bioavailability [8]. To address selenium deficiency, selenium-enriched foods containing organic selenium have been widely developed in recent years. Selenium-enriched foods mainly include selenium-enriched plants [9], animals [10], and microorganisms [11]. Numerous studies have demonstrated the health benefits of selenium-enriched foods in overcoming selenium deficiency [12,13]. Additionally, to fully establish the links between health benefits and specific selenium species, various analytical techniques have also been established for the analysis of total selenium content and its species in selenium-enriched foods [14]. However, to further advance the development of effective selenium-enriched foods and address selenium deficiency in humans, there is still some research progress that need to be summarized: (1) the metabolism pathways of the main selenium species present in foods, including Se(IV), Se(VI), SeCys, MeSeCys, SeMet, and SeCys_2_; (2) the biological functions of different selenium-enriched foods, rather than standard selenium; (3) accurate and sensitive analysis methods of total selenium and selenium species in selenium-enriched foods, which have serious matrix effects. This review will primarily focus on the metabolism of selenium, the physiological functions of selenium-enriched foods, and the analysis of selenium and its species in selenium-enriched foods.

## 2. Selenium and Human Health

Selenium, as one of the essential trace elements, significantly impacts the normal physiological metabolism of humans. It is taken in and accumulated in the human body through the daily diet.

### 2.1. Physiological Role of Selenium

Selenium, as an essential trace element, has a high nutritional value in the human body. Due to the low selenium content in the food chain, selenium deficiency can lead to various diseases such as Keshan disease [3], Kashin–Beck disease [3], myocardial infarction [4], Alzheimer’s disease [5], and chronic pancreatitis [1]. Conversely, excessive selenium intake also can lead to toxicity, resulting in symptoms like hair loss and skin lesions [15]. Recent studies have also shown that an excess of selenium may also cause type 2 diabetes [16,17] and serious intestinal diseases [18]. In extreme cases, acute poisoning can lead to heart attack, kidney failure, and even death [19]. As can be seen, both selenium deficiency and excess have negative effects on human health. The recommended dietary allowance for selenium intake depends on certain parameters like age, pregnancy, and breastfeeding, and Table 1 lists the daily intake of selenium recommended for different populations [20].

Within a narrow nutritional concentration range, selenium exhibits various biological activities in humans, including antioxidant and anticancer effects, detoxification, and others [21,22]. For example, selenium can act as an antioxidant by being synthesized into glutathione peroxidase (GPxs), which helps scavenge free radicals and protect cell membranes [23,24]. As an anticancer agent, selenium can be metabolized into selenocysteine, which inhibits protein synthesis, thereby suppressing cancer cell proliferation and causing cancer cell apoptosis [25]. Selenium, as a negatively charged nonmetallic ion, can also interact with positively charged metal ions (e.g., cadmium ions and mercury ions) to form metal–selenium–protein complexes, reducing the toxicity of metals [26]. Additionally, selenium can improve immunity by enhancing the bactericidal ability of macrophages [27]. Selenium has also been found to improve male fertility by increasing sperm concentration, motility, and seminal antioxidant capacity [28]. Lastly, a selenium-sufficient diet can prevent or treat some diseases such as cardiovascular and cerebrovascular diseases, Keshan disease, Kashin–Beck disease, and liver diseases [29].

### 2.2. Metabolism of Selenium

**Figure 1:**
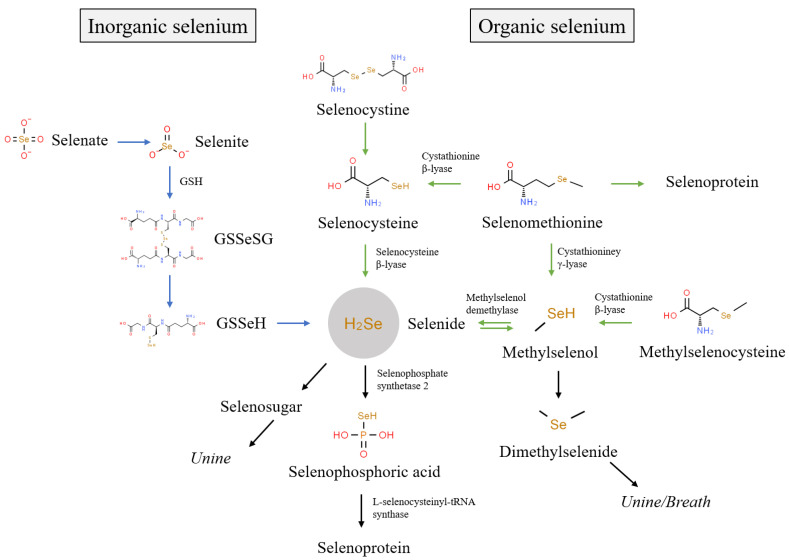
Metabolic pathways of different selenium species in organism.

The biological functions of selenium are primarily achieved through its metabolism into 25 selenoproteins in an organism [30]. The biological activity of selenium in foods depends upon its chemical species [8]. Generally, the existing species of selenium in foods are mainly divided into inorganic selenium (e.g., Se(IV) and Se(VI)) and organic selenium (e.g., SeCys_2_, MeSeCys, SeMet, and SeCys). Figure 1 shows the metabolism of different selenium species in organisms. Inorganic selenium, such as Se(IV) and Se(VI), is absorbed via passive diffusion and co-transport [31], while organic selenium with a higher absorption rate is absorbed via the active absorption pathway of amino acid [32]. In organisms, Se(VI) is reduced to Se(IV), which is further reduced into selenodiglutathione (GSSeSG), selenenylsulfide (GSSeH), and hydrogen selenide (H_2_Se) [31]. SeMet participates in the synthesis of selenium protein instead of methionine or is metabolized to SeCys via trans-sulfurization [33]. SeCys_2_ is reduced to SeCys by glutathione and glutathione reductase, and SeCys is converted to H_2_Se under the action of selenocysteine β-lyase [33,34]. In addition, MeSeCys is cleaved to methylselenol by cystathionine β-lyase, and further converted to H_2_Se under the demethylation reaction of methylselenol demethylase [35]. As can be seen, different selenium species are eventually metabolized to H_2_Se, which is involved in selenoprotein synthesis after activation to selenophosphoric acid [36]. The excess H_2_Se in tissues is further metabolized into methylselenol, dimethylselenide, and selenosugars, which are mainly excreted in urine and breath [33,34,35].

## 3. Development of Selenium-Enriched Foods

**Figure 2:**
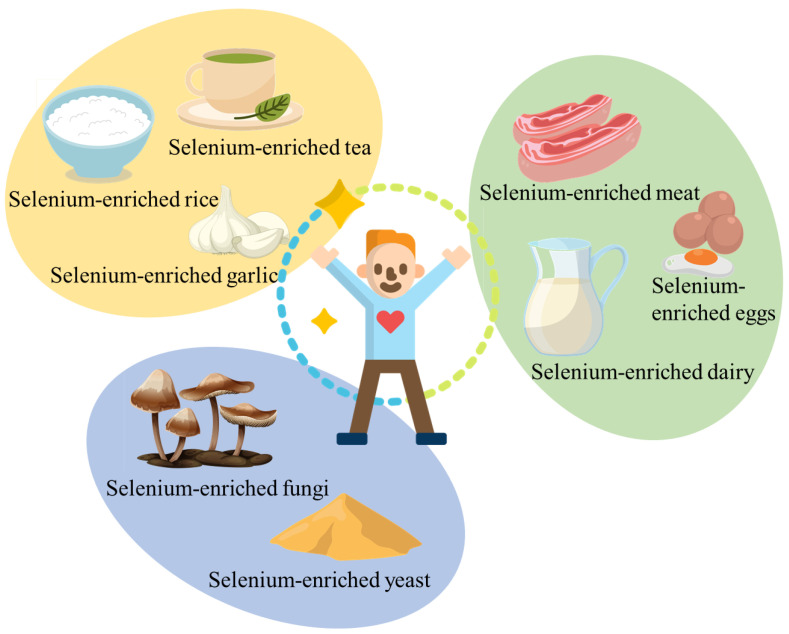
Different selenium-enriched foods.

Diet is the major source of selenium for the general population, and great attention has been paid to the development of various selenium-enriched foods to meet human nutritional needs. For example, according to the National Institutes of Health Office of Dietary Supplements, more than 30 food sources of selenium have been developed [37]. As shown in Figure 2, selenium-enriched foods are predominantly derived from plants (e.g., tea, rice, and garlic), animals (e.g., meat, eggs, and dairy), and microbials (e.g., yeast and fungi) in modern society. According to selenium enrichment methods, selenium-enriched foods can be categorized into natural selenium-enriched foods and artificial selenium-enriched foods, which are discussed in detail as follows.

### 3.1. Natural Selenium-Enriched Foods

Natural selenium-enriched foods are obtained from soil that naturally contains high levels of selenium, and inorganic selenium present in the soil can be transformed into various organic selenium species. Currently, several natural selenium-enriched foods, including selenium-enriched rice, tea, and garlic, have been obtained in selenium-enriched areas like Enshi in Hubei Province and Ziyang in Shanxi Province in China. However, it is worth noting that approximately 51% of China’s soil is deficient in selenium, and around 700 million people in China live in these selenium-deficient areas [38]. Consequently, relying solely on the consumption of natural selenium-enriched foods is not enough to meet the selenium supplementation needs of the population.

### 3.2. Artificial Selenium-Enriched Foods

Artificial selenium-enriched foods have been rapidly developed in the market, and mainly produced through plant transformation, animal transformation, and microorganism transformation. These methods efficiently and artificially transform the added inorganic selenium into different organic selenium species, ensuring the sufficient intake of selenium. Table 2 provides an overview of artificial selenium-enriched foods obtained by different methods.

#### 3.2.1. Plant Transformation of Selenium

Among the different approaches, plant transformation of selenium is the most effective approach to elevating daily selenium intake, and selenium-enriched plants are typically obtained through soil fertilization, foliar fertilization, and hydroponic fertilization [9,58]. Soil fertilization reduces the influence of environmental factors on plant growth. For example, selenium-enriched soybeans grown in soil supplemented with Na_2_SeO_3_ were obtained, and the selenium contents in beans, pods, leaves, and roots were found to be 75 ± 5, 16 ± 2, 36 ± 3, and 151 ± 14 μg/g, respectively. The predominant selenium species in beans were SeMet and SeCys, while the selenium species in other plant compartments was inorganic selenium [39]. Ebrahimi et al. [59] added Na_2_SeO_4_ or selenium-enriched plant residues into the soil to study the selenium biofortification in Brassica napus L, and confirmed that Na_2_SeO_4_ produced higher uptake efficiency. However, the bottlenecks of soil fertilization with selenium are low bioavailability and soil pollution.

Foliar fertilization with selenium can effectively reduce the loss of selenium during transport from the root to the stem and leaf, featuring high biofortification efficiency. For instance, Poblaciones et al. [40] found that the inorganic selenium added through foliar fertilization was efficiently absorbed by chickpeas, and mainly metabolited as SeMet (with the highest proportion, 91%) in chickpea. Mao et al. [60] obtained selenium-enriched Grifola frondosa polysaccharide by spraying Na_2_SeO_3_ during growth, and the selenium content was found to be 17.52 μg/g. However, foliar fertilization has drawbacks such as poor reproducibility and environmental pollution.

Compared with soil fertilization and foliar fertilization, hydroponic fertilization has advantages of simple operation, good reproducibility, and high absorption efficiency. Yu et al. [61] compared the uptake behavior of Na_2_SeO_3_ and Na_2_SeO_4_ in pak choi based on hydroponic fertilization, and revealed that Na_2_SeO_3_ was rapidly accumulated and transformed into MeSeCys. Similarly, Li et al. [41] also analyzed the uptake and transformation behaviors of Na_2_SeO_3_, Na_2_SeO_4_, and selenium nanoparticles in garlic through hydroponics, and found that Na_2_SeO_3_ and selenium nanoparticles were more easily metabolized into MeSeCys.

#### 3.2.2. Animal Transformation of Selenium

Selenium-enriched foods obtained through animal transformation mainly include selenium-enriched meat, selenium-enriched dairy, and selenium-enriched eggs. Selenium-enriched meat is obtained by feeding animals with selenium supplements and has been widely consumed by humans for selenium intake. Zhang et al. [10] treated pigs with different selenium species to obtain selenium-enriched pork, and found that SeMet and SeCys were the major selenium species in the meat. Mohamed et al. [62] fed chicks with Na_2_SeO_3_ and bacterial selenoprotein, and found that bacterial selenoprotein was more easily accumulated in the breast meat. Selenium-enriched dairy can be obtained by feeding animals (e.g., cows, goat, and sheep) with selenium. For example, Phipps et al. [50] fed cows with selenium yeast to obtain selenium-enriched dairy containing high levels of SeMet and SeCys. Additionally, selenium-enriched eggs are usually obtained by feeding chickens with selenium. For instance, Qiu et al. [63] fed laying hens with selenium-enriched insect protein, Na_2_SeO_3_, and selenium yeast, respectively. The results indicated that selenium-enriched insect protein had the best effects on hen growth and selenium content in eggs.

#### 3.2.3. Microbial Transformation of Selenium

As for microbial transformation, inorganic selenium added into the culture medium can interact with proteins or polysaccharides to produce selenium-enriched foods like selenium-enriched yeast and selenium-enriched fungi. These foods have emerged as valuable sources of selenium for humans in recent years. For example, selenium-enriched yeast was prepared by Na_2_SeO_3_ through fermentation accumulation, and the selenium content was found to be 14.95 mg/L [11]. Selenium-enriched auricularia cornea was produced by treating auricularia cornea with 100 mg/g Na_2_SeO_3_, and the genes involved in amino acid metabolism and lipid metabolism were up-regulated at the budding stage in response to selenium supplementation [64]. Likewise, selenium-enriched Lentinus edodes were cultivated in the medium containing 18.15 μg/g Na_2_SeO_3_, and the results indicated that selenium-enriched Lentinus edodes contained 0.2951 μg/g selenium and exhibited high antioxidant activity in RAW264.7 cells [65].

Overall, the development of selenium-enriched foods through different methods provides opportunities to meet the nutritional needs of the population. However, challenges such as low bioavailability and environmental pollution need to be addressed to ensure the effective and sustainable production of selenium-enriched foods.

## 4. Physiological Functions of Selenium-Enriched Foods

**Figure 3:**
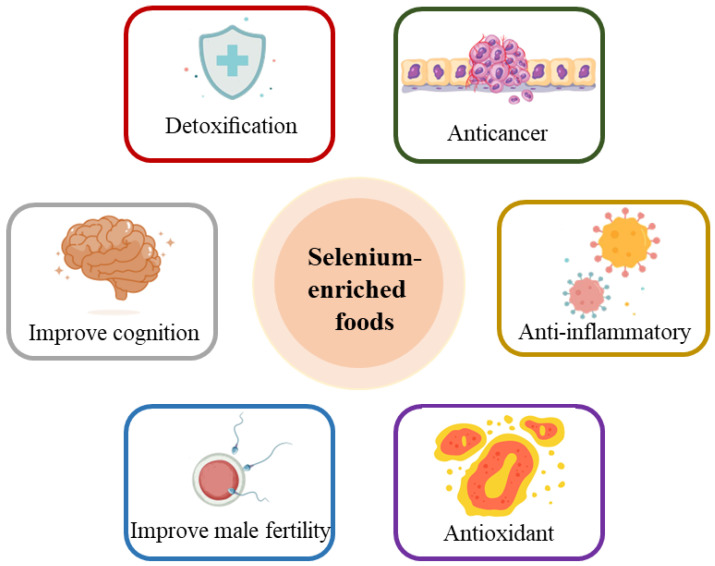
Physiological functions of selenium-enriched foods.

Several researchers have revealed that selenium combined with other active nutrients such as zinc and vitamin E in food could initiate synergistic health effects on various biological activities [12]. Figure 3 illustrates the diverse physiological functions of selenium-enriched foods, including antioxidant, anti-inflammatory, and anticancer effects; detoxification; improvement of male fertility; and others [13].

### 4.1. The Function of Reducing Oxidative Stress

In recent years, a variety of selenium-enriched foods have been confirmed to exhibit antioxidant properties. For example, Ma et al. [66] prepared selenium-enriched polysaccharides from Pleurotus ostreatus using hot water extraction, and the in vitro study results indicated that selenium-enriched polysaccharides exhibited high antioxidant capacity and reduced hydrogen peroxide-induced oxidative stress in murine skeletal muscle cells. Guo et al. [67] discovered that selenium-enriched yeast protein hydrolysate reduced ultraviolet B radiation-induced oxidative stress by increasing glutathione peroxidase and catalase activities in vivo. Li et al. [68] investigated the antioxidant effect of selenium-enriched G. frondosa on cyclophosphamide-treated mice, and found that selenium-enriched G. frondosa displayed stronger antioxidant activity through the MAPKs signaling pathways. Furthermore, healthy women supplemented with selenium-enriched rice experienced an increase in serum selenium levels and GPx-activity [69].

### 4.2. The Function of Inhibiting Inflammation

At present, selenium-enriched foods have been found to exert anti-inflammatory effects, mainly through the NF-κB/MAPKs signaling pathway. Chomchan et al. [70] found that selenium-enriched ricegrass juice extracts promoted macrophage cell proliferation and reduced nitric oxide levels in LPS-induced RAW264.7 cells, and the foremost bioactive components were identified as flavone glycosides by UHPLC-MS. Similarly, RAW264.7 cell assay also indicated that both selenium-enriched brown rice protein hydrolysates [71] and selenium-enriched oolong tea extract [72] exhibited excellent anti-inflammatory functions via the NF-κB/MAPKs signaling pathway. Furthermore, selenium-enriched Cordyceps militaris exhibited an anti-inflammatory effect in LPS-injured mice by inhibiting pro-inflammatory mediator production and increasing anti-inflammatory cytokine levels [73].

### 4.3. The Function of Inhibiting Cancer

Numerous medical studies have demonstrated the excellent anticancer activity of selenium-enriched foods. For instance, Zhang et al. [13] found that selenium-enriched polysaccharide fraction obtained by Pleurotus ostreatus induced the apoptosis of various cancer cells by inhibiting the epithelial-to-mesenchymal transition, without a significant effect on normal cells. Luo et al. [74] confirmed that selenium-enriched Cordyceps militaris inhibited the viability of NCI-H292 and A549 cells, and induced cancer cell apoptosis by altering the expression of apoptotic and cell cycle regulatory proteins. Daniela et al. [75] evaluated selenium-enriched chickpea sprouts and found that they inhibited cancer tumor growth through the overexpression of Fas protein in vivo.

### 4.4. The Function of Alleviating the Toxicity of Heavy Metals

Studies have indicated that selenium-enriched foods can alleviate the biological toxicity of heavy metals such as cadmium, mercury, and lead. For instance, Su et al. [76] found that selenium-enriched rice significantly alleviated injury in mice with cadmium poisoning by overexpressing antioxidant genes (e.g., Nrf-2, GPX1, TrxR2, and TNF-2). Shang et al. [77] elucidated that selenium-enriched probiotics had a detoxification effect on brain injury caused by cadmium poisoning through MAPK, calcium, and PI3K-Akt signaling pathways. Additionally, Shang et al. [78] demonstrated that selenium-enriched Bacillus subtilis protected carp from mercury-induced inflammation, effectively reducing mercury toxicity. Zhu et al. [79] investigated that selenium-enriched rice protein hydrolysates reduced lead-induced cytotoxicity via slowing the accumulation of lead in cells.

### 4.5. The Function of Improving Male Fertility

The ability of selenium-enriched foods to improve male fertility have also been reported. At the cellular level, a mouse testicular cell assay indicated that selenium-enriched green tea inhibited the chromosomal aberrations induced by mitomycin C [80]. As for animal experiments, selenium-enriched probiotic supplementation alleviated the adverse effects of hyperlipidemia in male mice by reducing testicular tissue damage, increasing serumal testosterone levels, and improving sperm indexes [81]. Selenium-enriched yeast also exhibited a significant effect on the improvement of male fertility of roosters [82]. Additionally, selenium-enriched Spirulina observably protected the reproductive systems of male zebrafish exposed to Beta-cypermethrin by enhancing antioxidant enzyme activity and androgen secretion [83]. These findings suggest that selenium-enriched foods may have potential benefits for improving male fertility in humans.

### 4.6. Other Functions

In addition to the aforementioned effects, selenium-enriched foods have other physiological effects such as improving cognition, regulating the balance of intestinal bacteria, and protecting the liver. For example, Yu et al. [84] found that the crude polysaccharides prepared from selenium-enriched C. militaris had positive antiobesity and gut microbiota modulatory effects. Jia et al. [85] found that selenium-enriched radish sprouts improved the antioxidant capacity and alleviated liver damage in mice treated with carbon tetrachloride.

## 5. Analysis of Total Selenium and Its Species in Selenium-Enriched Foods

Selenium-enriched foods have become the most convenient and effective method of supplementing selenium for humans. The nutritional value of selenium-enriched foods is not only related to the total selenium content, but also closely related to the species of selenium present in foods. Therefore, to provide insights on the development and nutritional analysis of high-quality selenium-enriched foods, it is necessary to analyze the total selenium and its species in selenium-enriched foods.

### 5.1. Analysis of Total Selenium

There are some quality problems in the produced selenium-rich foods, such as the total selenium content not being up to standards or exceeding standards. Therefore, analysis of total selenium content in selenium-enriched foods must be a higher priority to meet daily intakes. Prior to determining the total selenium content in selenium-enriched foods, it is necessary to pre-treat the samples with various digestion methods, including dry-ashing, wet digestion, and microwave digestion [86]. Then, the digested samples can be directly detected by different instruments, including hydride generation atomic fluorescence spectrometry (HG-AFS), molecular fluorescence spectroscopy (MFS), flame atomic absorption spectrometry (FAAS), and hydride generation inductively coupled plasma atomic emission spectrometry (HG-ICP-AES), etc. For example, Khan et al. [87] validated various digestion methods for analyzing the total selenium content in infant formulas combined with inductively coupled plasma optical emission spectrometry (ICP-OES) and inductively coupled plasma mass spectrometry (ICP-MS). The results confirmed that wet digestion and microwave methods exhibited better performance. The common analysis methods of total selenium content in selenium-enriched foods are listed in Table 3.

### 5.2. Analysis of Different Selenium Species

Since different selenium species have different bioavailabilities, metabolic pathways, and physiological effects in organisms, it is crucial to identify and quantify the different selenium species present in selenium-enriched foods. The analysis processes of selenium species mainly include sample pretreatment, separation of selenium species, and quantitative determination. The analysis methods of different selenium species in various foods based on ICP-MS are listed in Table 4.

#### 5.2.1. Sample Pretreatment

Before the analysis of different selenium species in selenium-enriched foods, it is necessary to extract various selenium species from the samples without specie transformation. The common extraction methods of selenium species in selenium-enriched foods mainly include water extraction [105], acid extraction [106], and enzymatic extraction [107]. Generally, water extraction is suitable for soluble selenium species such as Se(VI), Se(IV), MeSeCys, SeCys, SeCys_2_, SeMet, and GluMeSeCys. However, the recoveries of water extraction methods are low [97]. Acid extraction methods featuring good recovery can be used to extract selenium species incorporated into proteins, but may result in the degradation or transformation of selenium [106]. Compared to water extraction and acid extraction, enzymatic extraction has some advantages such as easier operation and reduced degradation of selenium [108]. In addition, specific methods such as solid-phase extraction [109] and liquid-phase microextraction [110] have also been established to extract selenium species.

#### 5.2.2. Separation and Detection of Different Selenium Species

After the extraction process, it is crucial to separate and detect different selenium species. Multiple techniques have been used for the separation of selenium species, including high-performance liquid chromatography (HPLC) [111], gas chromatography (GC) [112], and capillary electrophoresis (CE) [93]. Then, the separated selenium species can be quantitatively analyzed by a variety of detectors, including atomic fluorescence spectroscopy (AFS), atomic absorption spectroscopy (AAS), and ICP-MS [113,114,115]. Among them, ICP-MS, as the most effective method, has been widely used for selenium analysis in food samples due to its virtues of high sensitivity, wide dynamic range, and independence from the molecular structure.

Based on the separation and detection modes, we compared three types of method: CE-ICP-MS, GC-ICP-MS, and HPLC-ICP-MS. CE-ICP-MS has advantages such as lower sample consumption, short separation time, and simpler operation, and has been established for the analysis of selenium species. For example, Se(VI), Se(IV), MeSeCys, SeCys, and SeMet were separated by electrophoresis and detected by ICP-MS, with the limit of detection ranging from 0.5 to 1.4 ng/mL [103]. However, CE has the disadvantage of potential pipeline blockage due to the small tube diameter. Comparatively, GC-ICP-MS is mostly used for the analysis of volatile selenium species including methaselenol, dymethylselenide, and dimethyldiselenide, and the non-volatile selenium species need to be derivated. For instance, Yang et al. [116] developed a precise method to determine SeMet in yeast by GC-ICP-MS following derivatization with methyl chloroformate. The detection limit was 0.9 μg/g, and the content of SeMet in yeast was found to be 3434 ± 19 μg/g, which accounts for 67% of the total selenium. However, GC-ICP-MS methods are time-consuming for the derivatization and determination of non-volatile selenium species. Compared with GC and CE methods, HPLC is the most versatile separation technique with different separation modes (e.g., volume exclusion chromatography (SEC-HPLC) [117], ion exchange chromatography (IEC-HPLC) [118], and hydrophilic liquid-phase interaction chromatography (HILIC-HPLC) [119]. In addition, HPLC is easier to bring online coupled with ICP-MS for selenium species analysis. For instance, Cao et al. [120] established a sensitive method based on HPLC-ICP-MS to analyze elemental selenium, SeMet, SeCys_2_, and MeSeCys in selenium-enriched polysaccharide. The limits of detection for the four selenium species varied from 0.44 to 2.35 μg/L. In parallel, HPLC coupled with high-resolution instruments (e.g., electrospray ionization–mass spectrometry (ESI-MS, ESI-MS/MS) and time-of flight mass spectrometry (TOF-MS)) have also been widely developed for the accurate identification and analysis of the unknown species of selenium in selenium-enriched foods [121,122]. Overall, we believe that ICP-MS-based techniques are essential for analyzing selenium and its species in selenium-enriched foods, and beneficial to the monitoring of stability in the nutritional quality of selenium-enriched foods.

## 6. Conclusions and Future Perspectives

Selenium plays important roles in human health, but both selenium deficiency and excess can cause severe harm to humans and animals. Studies have shown that the effects of selenium supplementation depend on its dosage and species. Therefore, it is important to maintain a balanced and scientifical selenium supplementation. In recent years, natural and artificial selenium-enriched foods have been developed as the major sources of dietary selenium supplementation, and various selenium-enriched foods have been demonstrated to exhibit different physiological functions in vitro and in vivo, including antioxidant, anticancer, and anti-inflammatory effects, etc. In addition, different analysis methods based on ICP-MS have been established to determine the total selenium and its species in selenium-enriched foods, which is important to understand the bioaccumulation and metabolic pathways of selenium in these foods. To enhance the efficiency and safety of selenium supplementation, much effort should be devoted to the development of selenium-enriched foods in the future, and their physiological functions and mechanisms need to be further explored in clinical and preclinical studies.

## Figures and Tables

**Table 1:** Daily intake of selenium recommended for different populations [20].

Age	Selenium (μg/day)	
	Male	Female
0–4 months	10	10
4 months–4 years	15	15
4 years–7 years	20	20
7 years–10 years	30	30
10 years–13 years	45	45
13 years–15 years	60	60
15 years+	70	60
Pregnancy	-	60
Lactation	-	75

**Table 2:** Overview of artificial selenium-enriched foods obtained by different methods.

Selenium-Enriched Foods	Transformation Methods	Selenium Species	Ref.
Soybeans	Plant transformation	SeMet, SeCys	[39]
Chickpea	Plant transformation	Se(IV), Se(VI), SeMet	[40]
Garlic	Plant transformation	Se(IV), Se(VI), SeCys, MeSeCys	[41]
Wheat	Plant transformation	SeMet, SeCys	[42]
Potato	Plant transformation	SeMet	[43]
Broccoli	Plant transformation	SeMet, MeSeCys, SeCys_2_	[44]
Carrot	Plant transformation	SeMet	[45]
Astragalus	Plant transformation	SeMet, SeCys, MeSeCys	[46]
Brazilian nuts	Plant transformation	SeMet, SeCys	[47]
Pigs	Animal transformation	SeMet, SeCys, MeSeCys, selenourea	[10]
Sheep	Animal transformation	SeMet, SeCys	[48]
Chicken	Animal transformation	SeMet	[49]
Milk, cheese	Animal transformation	SeMet, SeCys	[50]
Tuna, mussel	Animal transformation	SeMet, trimethylselenonium ion	[51]
Shrimp	Animal transformation	Se(IV), Se(VI)	[52]
Atlantic salmon	Animal transformation	SeMet, SeCys	[53]
Egg	Animal transformation	SeMet, SeCys, MeSeCys	[54]
Yeast	Microorganism transformation	SeMet	[11]
Bailing mushroom	Microorganism transformation	Se(IV), Se(VI), SeMet, SeCys	[55]
Fungus	Microorganism transformation	Se(IV), SeMet, SeCys_2_	[56]
Agaricus bisporus	Microorganism transformation	MeSeCys	[57]

**Table 3:** Methods of total selenium analysis in selenium-enriched foods.

Scheme 0.	Digestion Methods	Detection Techniques	Selenium Content (mg/kg)	Ref.
Vegetable, pulses, and cereal	Dry-ashing	HG-AFS	0.01–0.204	[88]
Rice	Dry-ashing	HG-ICP-AES	0.201–0.53	[89]
Yeast	Wet digestion	FAAS	2145	[90]
Eggs	Wet digestion	HG-AFS	0.0961–0.152	[91]
Rapeseed	Wet digestion	MFS	0.052–3.52	[92]
Rice	Microwave digestion	ICP-OES	2.32	[93]
Rice, tea, and garlic	Microwave digestion	ICP-MS	0.0279–13.6	[94]

**Table 4:** Analysis of different selenium species in various foods by ICP-MS.

Selenium- Enriched Foods	Pre-Treatment Methods	Detection Techniques	Selenium Species	Ref.
Garlic	Water extraction	HPLC-ICP-MS	SeMet, MeSeCys, γ-glu-SeMeSeCys	[95]
Brassica juncea	Water extraction	GC-ICP-MS	Se(VI), Se(IV), SeCys, SeCys_2_, MeSeCys, CH_3_SeCH_3_, CH_3_SeSeCH_3_, CH_3_SeSCH_3_	[96]
Yeast	Enzyme extraction	HPLC-ICP-MS	SeMet, SeCys	[97]
Eggs	Enzyme extraction	HPLC-ICP-MS	Se(IV), SeMet	[98]
Rapeseed	Enzyme extraction	HPLC-ICP-MS	SeMet, SeCys_2_	[99]
Rice	Enzyme extraction	HPLC-ICP-MS	Se(IV), Se(VI), SeMet, SeCys_2_	[100]
Rice, tea, and garlic	Enzyme extraction	CE-ICP-MS	Se(IV), Se(VI), SeMet, SeCys_2_	[101]
Fish	Acid extraction	CE-ICP-MS	Se(IV), Se(VI), SeMet, SeCys_2_, MeSeCys	[102]
Yeast cells	Magnetic solid-phase extraction	HPLC-ICP-MS	SeMet, SeCys_2_, MeSeCys, selenoethionine, l-γ-glutamyl-Se-methyl-l-selenocysteine (GluMeSeCys)	[103]
Tea	Liquid–liquid microextraction	HPLC-ICP-MS	Se(IV), Se(VI)	[104]

## Data Availability

Not applicable.

## References

[1] Kieliszek M. (2019). Selenium-fascinating microelement, properties and sources in food. Molecules.

[2] Schweizer U., Fradejas-Villar N. (2016). Why 21? The significance of selenoproteins for human health revealed by inborn errors of metabolism. FASEB J..

[3] Hartikainen H. (2005). Biogeochemistry of selenium and its impact on food chain quality and human health. J. Trace Elem. Med. Biol..

[4] Shahid M., Niazi N.K., Khalid S., Murtaza B., Bibi I., Rashid M.I. (2018). A critical review of selenium biogeochemical behavior in soil-plant system with an inference to human health. Environ. Pollut..

[5] Pillai R., Uyehara-Lock J.H., Bellinger F.P. (2014). Selenium and selenoprotein function in brain disorders. IUBMB Life.

[6] Nothstein A.K., Eiche E., Riemann M., Nick P., Winkel L.H.E., Göttlicher J., Steininger R., Brendel R., Brasch M.V., Konrad G. (2016). Tracking Se assimilation and speciation through the rice plant-nutrient competition, toxicity and distribution. PLoS ONE.

[7] Wang N., Tan H., Li S., Xu Y., Guo W., Feng Y.B. (2017). Supplementation of micronutrient selenium in metabolic diseases: Its role as an antioxidant. Oxidative Med. Cell. Longev..

[8] Zhang K., Zhao Q.Y., Zhan T.F., Han Y.S., Tang C.H., Zhang J.M. (2020). Effect of different selenium sources on growth performance, tissue selenium content, meat quality, and selenoprotein gene expression in finishing pigs. Biol. Trace Elem. Res..

[9] Ari B., Oz E., Can S.Z., Bakirdere S. (2022). Bioaccessibility and bioavailability of selenium species in Se-enriched leeks (*Allium Porrum*) cultivated by hydroponically. Food Chem..

[10] Zhang K., Guo X.Q., Zhao Q.Y. (2020). Development and application of a HPLC-ICP-MS method to determine selenium speciation in muscle of pigs treated with different selenium supplements. Food Chem..

[11] Wu G.J., Liu F., Sun X.W., Lin X.G., Zhan F., Fu Z.H. (2019). Preparation of selenium-enriched yeast by re-using discarded saccharomyces cerevisiae from the beer industry for Se-supplemented fodder applications. Appl. Sci..

[12] Tangjaidee P., Swedlund P., Xiang J., Yin H.Q., Quek S.Y. (2022). Selenium-enriched plant foods: Selenium accumulation, speciation, and health functionality. Front. Nutr..

[13] Zhang Z.M., Zhang Y.S., Liu H., Wang J.H., Wang D., Deng Z.W., Li T.H., He Y., Yang Y.J., Zhong S.A. (2021). A water-soluble selenium-enriched polysaccharide produced by *Pleurotus ostreatus*: Purification, characterization, antioxidant and antitumor activities in vitro. Int. J. Biol. Macromol..

[14] Infante H.J., Hearn R., Catterick T. (2005). Current mass spectrometry strategies for selenium speciation in dietary sources of high-selenium. Anal. Bioanal. Chem..

[15] Navarro-Alarcon M., Cabrera-Vique C. (2008). Selenium in food and the human body: A review. Sci. Total Environ..

[16] Cardoso B.R., Braat S., Graham R.M. (2021). Selenium status is associated with insulin resistance markers in adults: Findings from the 2013 to 2018 national health and nutrition examination survey (NHANES). Front. Nutr..

[17] Casanova P., Monleon D. (2023). Role of selenium in type 2 diabetes, insulin resistance and insulin secretion. World J. Diabetes.

[18] Tortelly V.C., Melo D.F., Matsunaga A.M. (2018). The relevance of selenium to alopecias. Int. J. Trichol..

[19] Vinceti M., Mandrioli J., Borella P., Michalke B., Tsatsakis A., Finkelstein Y. (2014). Selenium neurotoxicity in humans: Bridging laboratory and epidemiologic studies. Toxicol. Lett..

[20] Kipp A.P., Strohm D., Brigelius-Flohe R., Schomburg L., Bechthold A., Leschik-Bonnet E., Heseker H. (2015). Revised reference values for selenium intake. J. Trace Elem. Med. Biol..

[21] Brigelius-Flohe R., Arner E.S.J. (2018). Selenium and selenoproteins in (redox) signaling, diseases, and animal models-200 year anniversary issue. Free Radic. Biol. Med..

[22] Wang L., Sagada G., Wang R.L., Li P.W., Xu B.Y., Zhang C., Qiao J.L., Yan Y.Z. (2022). Different forms of selenium supplementation in fish feed: The bioavailability, nutritional functions, and potential toxicity. Aquaculture.

[23] Zhang Y., Roh Y.J., Han S.J., Park I., Lee H.M., Ok Y.S., Lee B.C., Lee S.R. (2020). Role of selenoproteins in redox regulation of signaling and the antioxidant system: A review. Antioxidants.

[24] do Nascimento da Silva E., Aureli F., Amato M., Raggi A., Cadore S., Cubadda F. (2017). Selenium bioaccessibility and speciation in selenium-enriched lettuce: Investigation of the selenocompounds liberated after in vitro simulated human digestion using two-dimensional HPLC-ICP-MS. J. Agric. Food Chem..

[25] Gangadoo S., Stanley D., Hughes R.J., Moore R.J., Chapman J. (2017). The synthesis and characterisation of highly stable and reproducible selenium nanoparticles. Inorg. Nano-Met. Chem..

[26] Hasanuzzaman M., Hossain M.A., Fujita M. (2012). Exogenous selenium pretreatment protects rapeseed seedlings from cadmium-induced oxidative stress by upregulating antioxidant defense and methylglyoxal detoxification systems. Biol. Trace Elem. Res..

[27] Kursvietiene L., Mongirdiene A., Bernatoniene J., Sulinskiene J., Staneviciene I. (2020). Selenium anticancer properties and impact on cellular redox status. Antioxidants.

[28] Ghafarizadeh A.A., Vaezi G., Shariatzadeh M.A., Malekirad A.A. (2018). Effect of in vitro selenium supplementation on sperm quality in asthenoteratozoospermic men. Aadrologia.

[29] Han M.Q., Liu K.L. (2022). Selenium and selenoproteins: Their function and development of selenium-rich foods. Int. J. Food Sci. Technol..

[30] Nicholson J.L., Toh P., Alfulaij N., Berry M.J., Torres D.J. (2022). New insights on selenoproteins and neuronal function. Free Radic. Biol. Med..

[31] Thiry C., Ruttens A., Pussemier L., Schneider Y.J. (2013). An in vitro investigation of species-dependent intestinal transport of selenium and the impact of this process on selenium bioavailability. Br. J. Nutr..

[32] Longchamp M., Castrec-Rouelle M., Biron P., Bariac T. (2015). Variations in the accumulation, localization and rate of metabolization of selenium in mature Zea mays plants supplied with selenite or selenate. Food Chem..

[33] Schrauzer G. (2003). The nutritional significance, metabolism and toxicology of selenomethionine. Adv. Food Nutr. Res..

[34] Weekley C.M., Harris H.H. (2013). Which form is that? The importance of selenium speciation and metabolism in the prevention and treatment of disease. Chem. Soc. Rev..

[35] Mojadadi A., Au A., Salah W., Witting P., Ahmad G. (2021). Role for selenium in metabolic homeostasis and human reproduction. Nutrients.

[36] Lu J., Zhang J., Jiang C., Deng Y., Ozten N., Bosland M.C. (2016). Cancer chemoprevention research with selenium in the post-SELECT era: Promises and challenges. Nutr. Cancer.

[37] National Institutes of Health Office of Dietary Supplements (2021). Selenium. https://urldefense.com/v3/__https://ods.od.nih.gov/factsheets/Selenium-HealthProfessional__;!!Ls64Rlj6!2pE2fxi7c7Te0ptlS9FPiGZMKudk8S4dGMG5RF_bNtCjZlLWXdJNtI6WlLdKXJwqTf_5hDt5d2e8-1lgXxVs4A$.

[38] Dinh Q.T., Cui Z.W., Huang J., Tran T., Wang D., Yang W.X., Zhou F., Wang M.K., Yu D.S., Liang D.L. (2018). Selenium distribution in the Chinese environment and its relationship with human health: A review. Environ. Int..

[39] Chan Q., Afton S.E., Caruso J.A. (2010). Selenium speciation profiles in selenite-enriched soybean (Glycine Max) by HPLC-ICPMS and ESI-ITMS. Metallomics.

[40] Poblaciones M.J., Rodrigo S., Santamaria O., Chen Y., Mcgrath S.P. (2014). Selenium accumulation and speciation in biofortified chickpea (*Cicer arietinum* L.) under Mediterranean conditions. J. Sci. Food Agric..

[41] Li Y., Zhu N., Liang X., Zheng L.R., Zhang C.X., Li Y.F., Zhang Z.Y., Gao Y.X., Zhao J.T. (2020). A comparative study on the accumulation, translocation and transformation of selenite, selenate, and SeNPs in a hydroponic-plant system. Ecotoxicol. Environ. Saf..

[42] Wang M., Ali F., Wang M., Dinh Q.T., Zhou F., Banuelos G.S., Liang D.L. (2020). Understanding boosting selenium accumulation in Wheat (*Triticum aestivum* L.) following foliar selenium application at different stages, forms, and doses. Environ. Sci. Pollut. Res..

[43] Zhang H., Zhao Z., Zhang X., Zhang W., Huang L.Q., Zhang Z.Z., Yuan L.X., Liu X.W. (2019). Effects of foliar application of selenate and selenite at different growth stages on selenium accumulation and speciation in potato (*Solanum tuberosum* L.). Food Chem..

[44] Bodnar M., Konieczka P. (2016). Evaluation of candidate reference material obtained from selenium-enriched sprouts for the purpose of selenium speciation analysis. LWT-Food Sci. Technol..

[45] Kapolna E., Hillestrøm P.R., Laursen K.H., Husted S., Larsen E.H. (2009). Effect of foliar application of selenium on its uptake and speciation in carrot. Food Chem..

[46] Lindblom S.D., Valdez-Barillas J.R., Fakra S.C., Marcus M.A., Wangeline A.L., Pilon-Smits E.A.H. (2013). Influence of microbial associations on selenium localization and speciation in roots of Astragalus and Stanleya hyperaccumulators. Environ. Exp. Bot..

[47] Elidiane G.S., Lidiane R.V.M., Marco A.Z.A. (2013). Speciation analysis of selenium in plankton, Brazil nut and human urine samples by HPLC-ICP-MS. Talanta.

[48] Bierla K., Dernovics M., Vacchina V., Szpunar J., Bertin G., Lobinski R. (2008). Determination of selenocysteine and selenomethionine in edible animal tissues by 2D size-exclusion reversed-phase HPLC-ICP-MS following carbamidomethylation and proteolytic extraction. Anal. Bioanal. Chem..

[49] Cabanero A.L., Madrid Y., Camara C. (2005). Enzymatic probe sonication extraction of Se in animal-based food samples: A new perspective on sample preparation for total and Se speciation analysis. Anal. Bioanal. Chem..

[50] Phipps R.H., Grandison A.S., Jones A.K., Juniper D.I., Ramos E., Bertin G. (2008). Selenium supplementation of lactating dairy cows: Effects on milk production and total selenium content and speciation in blood, milk and cheese. Animals.

[51] Quijano M.A., Moreno P., Gutierrez A.M., Perez-Conde M.C., Camara C. (2000). Selenium speciation in animal tissues after enzymatic digestion by high-performance liquid chromatography coupled to inductively coupled plasma mass spectrometry. J. Mass Spectrom..

[52] Zheng Y., Wang Z.Y., Hou T.Y., Huang M.Q., Hao H.W. (2020). Determination of inorganic selenium in rice and shrimp by atomic fluorescence spectrometry with solid phase extraction separation. Chin. J. Anal. Lab..

[53] Sele V., Ornsrud R., Sloth J.J., Berntssen M.H.G., Amlund H. (2018). Selenium and selenium species in feeds and muscle tissue of Atlantic salmon. J. Trace Elem. Med. Biol..

[54] Zhao Y., Wang M., Yang M.R., Zhou J., Wang T.T. (2022). Determination of selenomethionine, selenocystine, and methylselenocysteine in egg sample by high performance liquid chromatography-inductively coupled plasma mass spectrometry. Separations.

[55] Zou Y., Du F., Zhang H., Hu Q. (2018). Selenium speciation and biological characteristics of selenium-rich Bailing mushroom, Pleurotus tuoliensis. Emir. J. Food Agric..

[56] Zhang J.J., Yu X.Q., Wei X.H., Hu T., Guo Y.B. (2020). Speciation analysis of selenium in selenium-enriched fungus. Chin. J. Anal. Lab..

[57] Prange A., Sari M., Ameln S., Hajdu C., Hambitzer R., Effinger S., Hormes J. (2019). Characterization of selenium speciation in selenium-enriched button mushrooms (*Agaricus bisporus*) and selenized yeasts (dietary supplement) using X-ray absorption near-edge structure (XANES) spectroscopy. J. Trace Elem. Med. Biol..

[58] Schiavon M., Berto C., Malagoli M., Trentin A., Sambo P., Dall’Acqua S., Pilon-Smits E. (2016). Selenium biofortification in radish enhances nutritional quality via accumulation of methyl-selenocysteine and promotion of transcripts and metabolites related to glucosinolates, phenolics, and amino Acids. Front. Plant Sci..

[59] Ebrahimi N., Hartikainen H., Hajiboland R., Seppanen M.M. (2019). Uptake and remobilization of selenium in *Brassica napus* L. plants supplied with selenate or selenium-enriched plant residues. J. Plant Nutr. Soil Sci..

[60] Mao G.H., Zou Y., Feng W.W. (2014). Extraction, preliminary characterization and antioxidant activity of Se-enriched Maitake polysaccharide. Carbohyd. Polym..

[61] Yu Y., Liu Z., Luo L.Y., Fu P.N., Wang Q., Li H.F. (2019). Selenium uptake and biotransformation in Brassica rapa supplied with selenite and selenate: A hydroponic work with HPLC speciation and RNA-sequencing. J. Agric. Food Chem..

[62] Mohamed D.A., Sazili A.Q., Chwen L.T. (2020). Effect of microbiota-selenoprotein on meat selenium content and meat quality of broiler chickens. Animals.

[63] Qiu K., Zheng J.J., Obianwuna U.E. (2021). Effects of dietary selenium sources on physiological status of laying hens and production of selenium-enriched eggs. Front. Nutr..

[64] Li X.L., Yan L.J., Li Q. (2019). Transcriptional profiling of Auricularia cornea in selenium accumulation. Sci. Rep..

[65] Gu H.F., Liang L., Zhu X.P. (2023). Optimization of enzymatic extraction, characterization and bioactivities of Se-polysaccharides from Se-enriched Lentinus edodes. Food Biosci..

[66] Ma L., Zhao Y., Yu J., Ji H.Y., Liu A.J. (2018). Characterization of se-enriched Pleurotus ostreatus polysaccharides and their antioxidant effects in vitro. Int. J. Biol. Macromol..

[67] Guo H.K., Guo S.Y., Liu H.M. (2020). Antioxidant activity and inhibition of ultraviolet radiation-induced skin damage of Selenium-rich peptide fraction from selenium-rich yeast protein hydrolysate. Bioorg. Chem..

[68] Li Q., Chen G., Chen H., Zhang W.J., Ding Y.Y., Yu P., Zhao T., Mao G.H., Feng W.W., Yang L.Q. (2018). Se-enriched G. frondosa polysaccharide protects against immunosuppression in cyclophosphamide-induced mice via MAPKs signal transduction pathway. Carbohydr. Polym..

[69] Giacosa A., Faliva M.A., Perna S., Minoia C., Ronchi A., Rondanelli M. (2014). Selenium fortification of an Italian rice cultivar via foliar fertilization with sodium selenate and its effects on human serum selenium levels and on erythrocyte glutathione peroxidase activity. Nutrients.

[70] Chomchan R., Puttarak P., Brantner A., Siripongvutikorn S. (2018). Selenium-rich ricegrass juice improves antioxidant properties and nitric oxide inhibition in macrophage cells. Antioxidants.

[71] Feng M.J., Wang X.Y., Xiong H., Qiu T.T., Zhang H., Guo F.H., Jiang L., Sun Y. (2021). Anti-inflammatory effects of three selenium-enriched brown rice protein hydrolysates in LPS-induced RAW264.7 macro phages via NF-kB/MAPKs signaling pathways. J. Funct. Foods.

[72] Wang Q., Huang J.Q., Zheng Y.F., Guan X.F., Lai C.C., Gao H.Y., Ho C.T., Lin B. (2022). Selenium enriched oolong tea (*Camellia sinensis*) extract exerts anti inflammatory potential via targeting NF-kB and MAPK pathways in macrophages. Food Sci. Hum. Well..

[73] Wu S.J., Wu Q.P., Wang J., Li Y.F., Chen B., Zhu Z.J., Huang R., Chen M.F., Huang A.H., Xie Y.Z. (2022). Novel selenium peptides obtained from selenium-enriched cordyceps militaris alleviate neuroinflammation and gut microbiota dysbacteriosis in LPS-injured mice. J. Agric. Food Chem..

[74] Luo L., Ran R., Yao J., Zhang F., Xing M.H., Jin M., Wang L.Q., Zhang T. (2019). Se-enriched cordyceps militaris inhibits cell proliferation, induces cell apoptosis, and causes G2/M phase arrest in human non-small cell lung cancer cells. Oncotargets Ther..

[75] Guardado-Felix D., Antunes-Ricardo M., Rocha-Pizana M.R., Martinez-Torres A.C., Gutierrez-Uribe J.A., Saldivar S. (2019). Chickpea (*Cicer arietinum* L.) sprouts containing supranutritional levels of selenium decrease tumor growth of colon cancer cells xenografted in immune-suppressed mice. J. Funct. Foods.

[76] Su Y., Li L., Farooq M.U., Huang X., Zheng T.D., Zhang Y.J., Ei H.H., Panhwar F.H., Tang Z.C., Zeng R. (2021). Rescue effects of Se-enriched rice on physiological and biochemical characteristics in cadmium poisoning mice. Environ. Sci. Pollut. Res..

[77] Shang X., Geng L., Zhao Z., Luo L., Shi X.D., Zhang Q., Du R.J., Cong T.F., Xu W. (2022). Transcriptomics reveals the mechanism of selenium-enriched *Lactobacillus plantarum* alleviating brain oxidative stress under cadmium stress in *Luciobarbus capito*. Ecotoxicol. Environ. Saf..

[78] Shang X.C., Wang B., Sun Q.S., Zhang Y., Lu Y.T., Liu S.J., Li Y.H. (2022). Selenium-enriched *Bacillus subtilis* reduces the effects of mercury-induced on inflammation and intestinal microbes in carp (*Cyprinus carpio* var. *specularis*). Fish Physiol. Biochem..

[79] Zhu Y.Q., Ding J., Shi Y., Fang Y., Li P., Fan F.J., Wu J., Hu Q.H. (2021). Deciphering the role of selenium-enriched rice protein hydrolysates in the regulation of Pb^2+^-induced cytotoxicity: Anin vitroCaco-2 cell model study. Int. J. Food Sci. Technol..

[80] Li F., Xu J., Zhou J., Zhao L.Y., Sheng J.C., Sun G.J., Hu Q.H. (2009). Inhibition of mitomycin C-induced chromosomal aberrations by micrometer powder of selenium-enriched green tea in mice spermatocytes. Mutat. Res. Genet. Toxicol. Environ..

[81] Ibrahim H., Zhu Y., Wu C., Lu C.H., Ezekwe M.O., Liao S.F., Haung K.H. (2012). Selenium-enriched probiotics improves murine male fertility compromised by high fat diet. Biol. Trace Elem. Res..

[82] Razieh S., Ahmad Z., Mahdi Z., Yousefi A.R., Rafieian H.R. (2022). Effects of dietary supplementation of different sources and levels of selenium on the semen quality and reproductive performance in aged broiler breeder roosters. Poultry Sci..

[83] Zhang Y., Zhou Y., Tang Q., Hu F., Feng L.X., Shen J.L., Huang B. (2018). The protective effects of selenium-enriched spirulina on the reproductive system of male zebrafish (*Danio rerio*) exposed to beta-cypermethrin. Food Funct..

[84] Yu M., Yue J., Hui N., Zhi Y.E., Hayat K., Yang X.J., Zhang D., Chu S.H., Zhou P. (2021). Anti-hyperlipidemia and gut microbiota community regulation effects of selenium-rich *Cordyceps militaris* Polysaccharides on the high-fat diet-fed mice model. Foods.

[85] Jia L., Wang T., Sun Y., Zhang M.R., Tian J.Y., Chen H., Shen Z.G., Abro H.K., Su N.N., Cui J. (2019). Protective effect of selenium-enriched red radish sprouts on carbon tetrachloride-induced liver injury in mice. J. Food Sci..

[86] Ducros V., Ruffieux D., Belin N., Favier A. (1994). Comparison of two digestion methods for the determination of selenium in biological samples. Analyst.

[87] Khan N., Jeong I.S., Hwang I.M., Kim J.S., Choi S.H., Nho E.Y., Choi J.Y., Kwak B.M., Ahn J.H., Yoon T. (2013). Method validation for simultaneous determination of chromium, molybdenum and selenium in infant formulas by ICP-OES and ICP-MS. Food Chem..

[88] Matos-Reyes M.N., Cervera M.L., Campos R.C., de la Guardia M. (2010). Total content of As, Sb, Se, Te and Bi in Spanish vegetables, cereals and pulses and estimation of the contribution of these foods to the Mediterranean daily intake of trace elements. Food Chem..

[89] Monica P., Luigi F., Patrizia M., Giangiacomo B., Roberto M. (2007). Determination of selenium in Italian rices by differential pulse cathodic stripping voltammetry. Food Chem..

[90] Cathal D., Connolly R.F., Michael H. (2004). Validation of method for total selenium determination in yeast by flame atomic absorption spectrometry. Biol. Trace Elem. Res..

[91] Sun H.W., Feng B. (2011). Speciation of organic and inorganic selenium in selenium-enriched eggs by hydride generation atomic fluorescence spectrometry. Food Anal. Methods.

[92] Zhang H.J., Liu D.C. (2006). Determination of selenium in rapeseed by fluorometric method. China Oils Fats.

[93] Grotti M., Lagomarsino C., Magi E. (2006). Simultaneous determination of arsenic, selenium and mercury in foodstuffs by chemical vapour generation inductively coupled plasma optical emission spectroscopy. Ann. Chim..

[94] Chen S.Z., Liu L.P., Tang D.J. (2021). Determination of total and inorganic selenium in selenium-enriched rice, tea, and garlic by high-performance liquid chromatography-inductively coupled plasma mass spectrometry (HPLC-ICP-MS). Anal. Lett..

[95] Dumont E., Ogra Y., Vanhaecke F., Suzuki K.T., Cornelis R. (2006). Liquid chromatography-mass spectrometry (LC-MS): A powerful combination for selenium speciation in garlic (*Allium sativum*). Anal. Bioanal. Chem..

[96] Mounicou S., Shah M., Meija J., Caruso J.A., Vonderheide A.P., Shann J. (2006). Localization and speciation of selenium and mercury in *Brassica juncea*-implications for Se-Hg antagonism. J. Anal. Atom. Spectrom..

[97] Jagtap R., Maher W. (2016). Determination of selenium species in biota with an emphasis on animal tissues by HPLC-ICP-MS. Microchem. J..

[98] Bhatia P., Aureli F.D., Amato M., Prakash R., Cameotra S., Nagaraja T.P., Cubadda F. (2013). Selenium bioaccessibility and speciation in biofortifed Pleurotus mushrooms grown on selenium-rich agricultural residues. Food Chem..

[99] Moreda J., Sánchez J., Mañana A., Turnes I., Alonso E., López P., Muniategui S. (2018). Selenium species determination in foods harvested in Seleniferous soils by HPLC-ICP-MS after enzymatic hydrolysis assisted by pressurization and microwave energy. Food Res. Int..

[100] Gao H.H., Chen M.X., Hu X.Q., Chai S.S., Qin M.L., Cao Z.Y. (2018). Separation of selenium species and their sensitive determination in rice samples by ion-pairing reversed-phase liquid chromatography with inductively coupled plasma tandem mass spectrometry. J. Sep. Sci..

[101] Yun Q.Z., Zheng J.P., Yang M.W., Yang G.D., Wu Y.N., Fu F.F. (2011). Speciation analysis of selenium in rice samples by using capillary electrophoresis-inductively coupled plasma mass spectrometry. Talanta.

[102] Hsieh M.W., Liu C.L., Chen J.H., Jiang S.J. (2010). Speciation analysis of arsenic and selenium compounds by CE-dynamic reaction cell-ICP-MS. Electrophoresis.

[103] Chen B.B., Hu B., He M., Huang Q., Zhang Y., Zhang X. (2013). Speciation of selenium in cells by HPLC-ICP-MS after (on-chip) magnetic solid phase extraction. J. Anal. Atom. Spectrom..

[104] Zhou Q.X., Lei M., Li J., Wang M.Y., Zhao D.C., Xing A., Zhao K.F. (2015). Selenium speciation in tea by dispersive liquid-liquid microextraction coupled to high-performance liquid chromatography after derivatization with 2,3-diaminonaphthalene. J. Sep. Sci..

[105] Both E.B., Stonehouse G.C., Lima L.W., Fakra S.C., Aguirre B., Wangeline A.L., Xiang J.Q., Yin H.Q., Jokai Z., Soos A. (2020). Selenium tolerance, accumulation, localization and speciation in a Cardamine hyperaccumulator and a non-hyperaccumulator. Sci. Total Environ..

[106] Cuderman P., Ožbolt L., Kreft I., Stibilj V. (2010). Extraction of Se species in buckwheat sprouts grown from seeds soaked in various Se solutions. Food Chem..

[107] Pyrzynska K., Sentkowska A. (2019). Liquid chromatographic analysis of selenium species in plant materials. Trends Anal. Chem..

[108] Shao S.X., Mi X.B., Ouerdane L., Lobinski P., Garcia-Reyes J.F., Molina A., Vass A., Dernovics M. (2013). Quantification of Se-methylselenocysteine and its γ-glutamyl derivative from naturally Se-enriched green bean (*Phaseolus vulgaris vulgaris*) after HPLC-ESI-TOF-MS and Orbitrap MSn-based identification. Food Anal. Method.

[109] Acikkapi A.N., Tuzen M., Hazer B. (2019). A newly synthesized graft copolymer for magnetic solid phase microextraction of total selenium and its electrothermal atomic absorption spectrometric determination in food and water samples. Food Chem..

[110] Altunay N., Elik A., Kaya S. (2020). Alcohol-DES based vortex assisted homogenous liquid-liquid microextraction approach for the determination of total selenium in food samples by hydride generation AAS: Insights from theoretical and experimental studies. Talanta.

[111] Lenz M., Floor G.H., Winkel L.H.E., Roman G., Corvini P. (2012). Online preconcentration-IC-ICP-MS for selenium quantification and speciation at Ultratraces. Environ. Sci. Technol..

[112] Gionfriddo E., Naccarato A. (2012). A reliable solid phase micro extraction-gas chromatography-triple quadrupole mass spectrometry method for the assay of selenome-thionine and selenomethyl selenocysteine in aqueous extracts: Difference between selenized and not-enriched selenium potatoes. Anal. Chim. Acta.

[113] Yan L., Deng B., Shen C., Long C.J., Deng Q.F., Tao C.Y. (2015). Selenium speciation using capillary electrophoresis coupled with modified electrothermal atomic absorption spectrometry after selective extraction with 5-sulfosalicylic acid functionalized magnetic nanoparticles. J. Chromatogra. A.

[114] Tuzen M., Pekiner O.Z. (2015). Ultrasound-assisted ionic liquid dispersive liquid-liquid microextraction combined with graphite furnace atomic absorption spectrometric for selenium speciation in foods and beverages. Food Chem..

[115] Mazej D., Falnoga I., Veber M., Stibilj V. (2006). Determination of selenium species in plant leaves by HPLC-UV-HG-AFS. Talanta.

[116] Yang L., Sturgeon R.E., Wolf W.R., Goldschmidt R.J., Mester Z. (2004). Determination of selenomethionine in yeast using CNBr derivatization and species specific isotope dilution GC ICP-MS and GC-MS. J. Anal. Atom. Spectrom..

[117] Bierła K., Suzuki N., Ogra Y., Szpunar J., Lobinski R. (2017). Identification and determination of selenohomolanthionine-The major selenium compound in Torula yeast. Food Chem..

[118] Hu T., Hui G.F., Li H.F., Guo Y.B. (2020). Selenium biofortification in Hericium erinaceus (Lion’s Mane mushroom) and its in vitro bioaccessibility. Food Chem..

[119] Arnaudguilhem C., Bierła K., Ouerdane L., Preud H., Yiannikouris A., Lobinski R. (2012). Selenium metabolomics in yeast using complementary reversed-phase/hydrophilic ion interaction (HILIC) liquid chromatography-electrospray hybrid quadrupole trap/Orbitrap mass spectrometry. Anal. Chim. Acta.

[120] Cao J.P., Cheng Y.Z., Xu B.C., Wang Y.Z., Wang F.Q. (2021). Determination of different selenium species in selenium-enriched polysaccharide by HPLC-ICP-MS. Food Anal. Method.

[121] Ouerdane L., Both E.B., Xiang J., Yin H.Q., Kang Y., Shao S.X., Kiszelak K., Jokai Z., Dernovics M. (2020). Water soluble selenometabolome of *Cardamine violifolia*. Metallomics.

[122] Gilbert-López B., Dernovics M., Moreno-González D., Molina-Diaz A., Garcia-Reyes J.F. (2017). Detection of over 100 selenium metabolites in selenized yeast by liquid chromatography electrospray time-of-flight mass spectrometry. J. Chromatogr. B.

